# Supplementary material for: Administration of Slow-Release Synthetic Prostacyclin Agonist Promoted Angiogenesis and Skeletal Muscle Regeneration for Limb Ischemia
Source: Mol Ther Methods Clin Dev. 2020 May 22;18:119–30. doi: 10.1016/j.omtm.2020.05.022 (PMC7321796; doi:10.1016/j.omtm.2020.05.022)

OMTM, Volume *18*

Supplemental Information

Administration of Slow-Release Synthetic Prostacyclin Agonist Promoted Angiogenesis and Skeletal Muscle Regeneration for Limb Ischemia

Takaya Nakagawa, Shigeru Miyagawa, Takashi Shibuya, Yoshiki Sakai, Akima Harada, Kenichi Watanabe, and Yoshiki Sawa

Supplement data 1

Sline	preoperation	postoperation	1 week	2 week	3 week	4 week
①	95.9	9.9	21.6	31	26.3	36.1
②	116.9	8	12.0	21.8	29	31.4
③	85.9	8.2	22.9	28.4	25.2	30.5
④	87.1	3.3	7.59	23.9	25.2	26
⑤	91.6	5.5	7.0	15.1	33.8	32.8
⑥	86.5	5.76	19.6	21.3	26.4	29
⑦	111.4	3.11	13.6	37.6	38.4	38.6
⑧	107.1	6.58	13.1	22.5	35.2	33.6
⑨	127.5	7.88	12.4	21.15	25.5	26.3
⑩	97.6	3.3	8.69	23	24.9	27.1
average	100.75	6.153	13.848	24.575	28.99	31.14
standard deviation	13.62793088	2.25668806	5.42137584	5.939160294	4.70987261	4.003548426

Supplement data 2

YS-1402	preoperation	postoperation	1 week	2 week	3 week	4 week
①	105.3	4.19	10.9	29.5	36.7	43.8
②	101.2	4.4	17.7	23.9	35.8	45.9
③	81.6	4.2	11.0	13.2	68.7	75.1
④	79	9.57	21.5	42.5	43.2	50.4
⑤	94.8	3.91	3.14	16.7	35.7	43.1
⑥	128	5.18	27	32.5	49.1	63
⑦	135	4.14	14.2	18.96	33.3	51.7
⑧	100	2.82	6.66	25.3	32.1	46.7
⑨	91.3	4.05	17.9	56.6	71.7	81.9
average	101.8	4.717777778	14.44444444	28.79555556	45.14444444	55.73333333
standard deviation	17.92471912	1.80859539	7.014567558	12.89946348	14.28970967	13.48900787

Supplement data 3

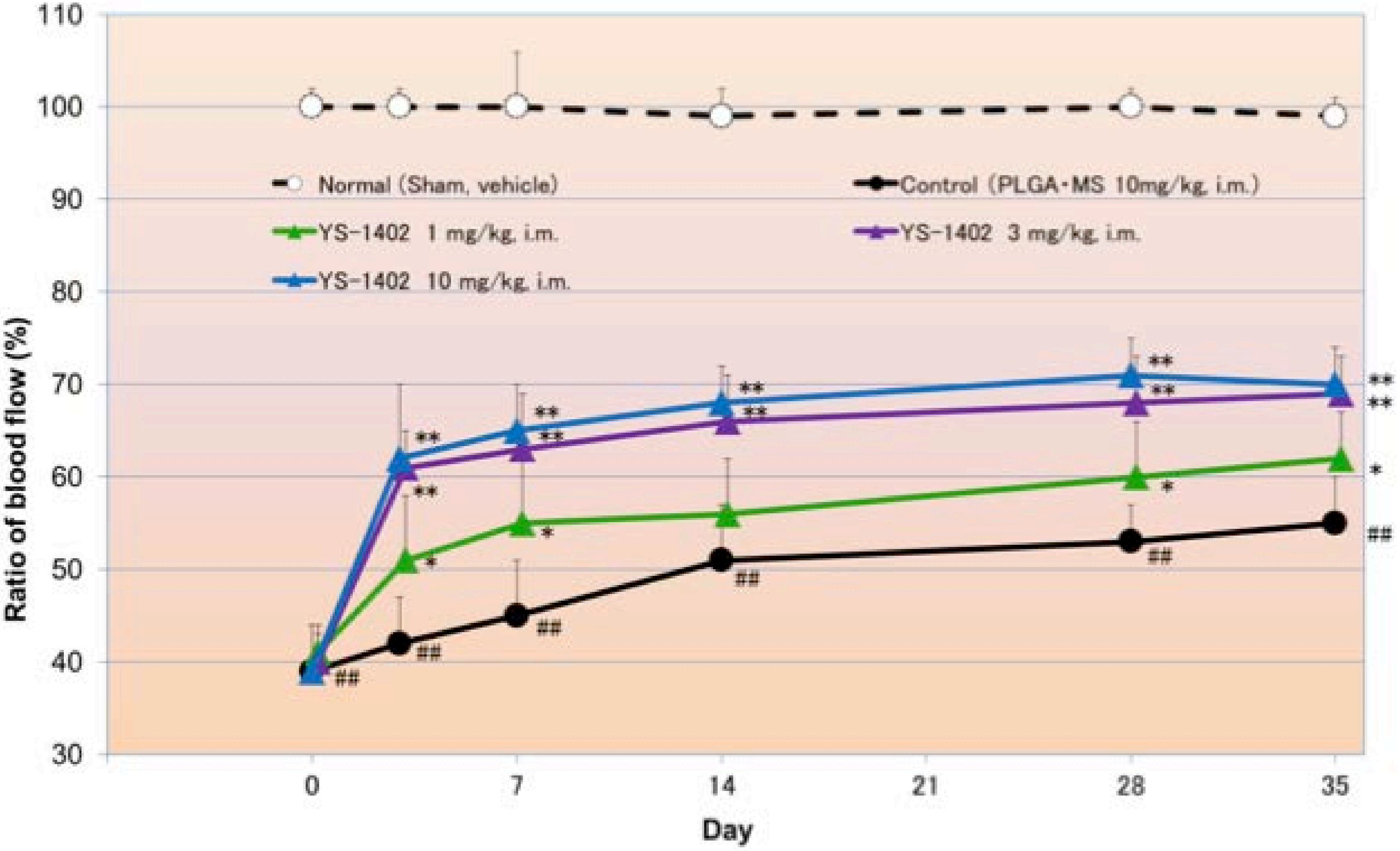

Supplement data 4

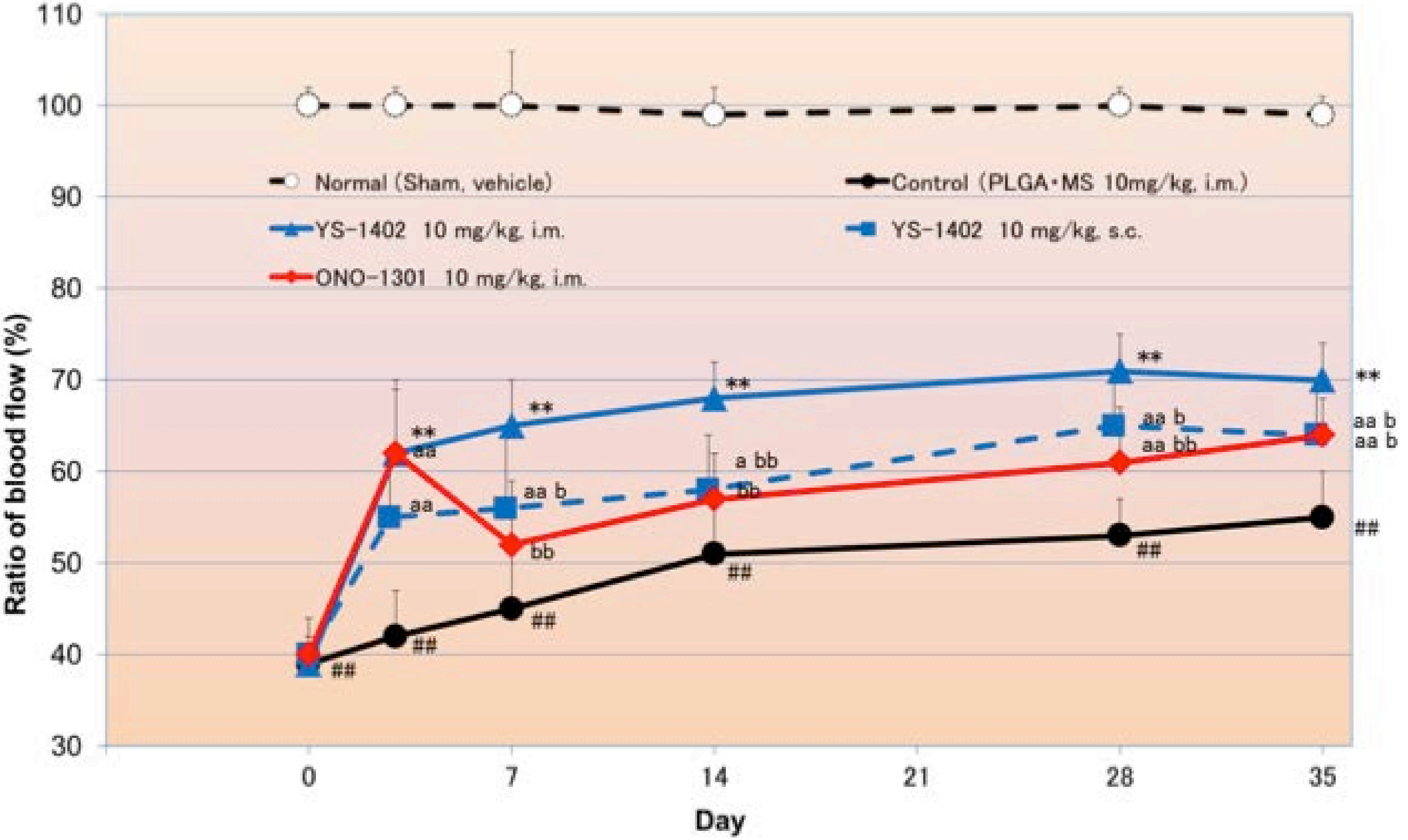

Supplement: Document S1. Data S1–S4 [file mmc1.pdf]
