# Supplementary material for: Administration of Slow-Release Synthetic Prostacyclin Agonist Promoted Angiogenesis and Skeletal Muscle Regeneration for Limb Ischemia
Source: Mol Ther Methods Clin Dev. 2020 May 22;18:119–30. doi: 10.1016/j.omtm.2020.05.022 (PMC7321796; doi:10.1016/j.omtm.2020.05.022)

Molecular Therapy
Methods & Clinical Development
Original Article

Administration of Slow-Release Synthetic Prostacyclin Agonist Promoted Angiogenesis and Skeletal Muscle Regeneration for Limb Ischemia

Takaya Nakagawa,[1] Shigeru Miyagawa,[1] Takashi Shibuya,[1] Yoshiki Sakai,[1] Akima Harada,[1] Kenichi Watanabe,[1] and Yoshiki Sawa[1]

[1]Department of Cardiovascular Surgery, Osaka University Graduate School of Medicine, Suita, Osaka 565-0871, Japan

Gene or cell therapy is currently not fully efficacious for arteriosclerosis obliterans (ASO). In this study, we determined whether YS-1402, a slow-release synthetic prostacyclin agonist, promoted neovascularization and skeletal muscle regeneration in a mouse model of critical limb ischemia (CLI). We ligated the femoral artery and its branches to obtain the CLI mouse model, administered saline (S group) or YS-1402 (YS group) to the thigh adductor 1 week after femoral artery occlusion, and evaluated tissue blood flow after surgery. After treatment, the leg muscle was obtained for histological, gene expression, and protein analyses to assess angiogenesis and skeletal muscle regeneration. Tissue blood flow improved in the YS group compared with that in the S group, and the number of CD31$^+$/α-smooth muscle actin (αSMA)$^+$ arterioles increased in the YS group. Prostacyclin receptor (IPR), stromal cell-derived factor-1, hepatocyte growth factor, and neural cell adhesion molecule expression levels were higher in the YS than in the S group. Skeletal muscle regeneration was detected based on PAX7- and Ki-67-positive satellite cells in the YS group. Myogenin and MyoD expression was higher in the YS than in the S group. Therefore, YS-1402 promoted functional angiogenesis and skeletal muscle regeneration in the CLI mouse model, suggesting a new therapy for ASO.

INTRODUCTION

Critical limb ischemia (CLI) is a peripheral artery disease that results in severe blockage of the arteries of the lower extremities, has a high mortality and limb amputation rates, and usually has a poor prognosis.[1–4] Currently, oral medications, bypass surgeries, and endovascular treatments are used for treatment. However, there is no indication for revascularization in approximately 25%–40% of CLI patients, and sufficient treatment has not been established.[2] Therefore, development of a new treatment method is required.

Revascularization therapy for peripheral vascular diseases includes gene therapy (such as vascular endothelial growth factor [VEGF]), autologous stem cell therapy (such as autologous bone marrow hepatocyte transplantation), and cytokine therapy (such as granulocyte colony-stimulating factor).[5–9] However, none of these therapies has been established to be safe and efficient for treatment.[10–12] The main cause of CLI is ischemia resulting from the disruption of blood vessels that leads to muscle damage; thus, it is critical to establish efficient therapeutic methods that target both angiogenesis and tissue regeneration in the decaying skeletal muscle for complete repair of the damaged limb.[13]

We are developing a new type of prostacyclin agonist, ONO-1301, which is also a prostaglandin receptor agonist and thromboxane A2 synthase inhibitor, and constructing a more effective poly(lactic-co-glycolic acid) (PLGA)-encapsulating drug delivery system that can release prostacyclin agonist for 4 weeks.[14–23]

ONO-1301 increases intracellular cyclic adenosine monophosphate by binding to a specific receptor—prostacyclin receptor (IPR)—in vascular endothelial and smooth muscle cells, and by releasing various protective cytokines and chemokines, such as VEGF, hepatocyte growth factor (HGF), and stromal cell-derived factor-1 (SDF-1). It enhances the expression of these genes and is expected to have a beneficial effect in the peripheral blood vessel region for muscle regeneration.

Therefore, we hypothesized that the slow-release synthetic prostacyclin agonist ONO-1301 (YS-1402), which is a cell-free regenerative medicine that induces the expression of various cytokines, promotes neovascularization and skeletal muscle regeneration in a CLI mouse model. This study sought to analyze the efficacy of YS-1402 in a CLI mouse model (see Figure 1 for the study protocol).

RESULTS

Increased Blood Perfusion after YS-1402 Administration

One week after treatment, laser Doppler perfusion imaging (LDPI) revealed significant improvement in tissue blood flow in the YS group (group administered YS-1402) compared with that in the

Received 6 February 2020; accepted 19 May 2020;
https://doi.org/10.1016/j.omtm.2020.05.022.

Correspondence: Yoshiki Sawa, MD, PhD, Department of Cardiovascular Surgery, Osaka University Graduate School of Medicine, 2-2 Yamadaoka, Suita, Osaka 565-0871, Japan.
E-mail: sawa-p@surg1.med.osaka-u.ac.jp

This is an open access article under the CC BY license (http://creativecommons.org/licenses/by/4.0/).

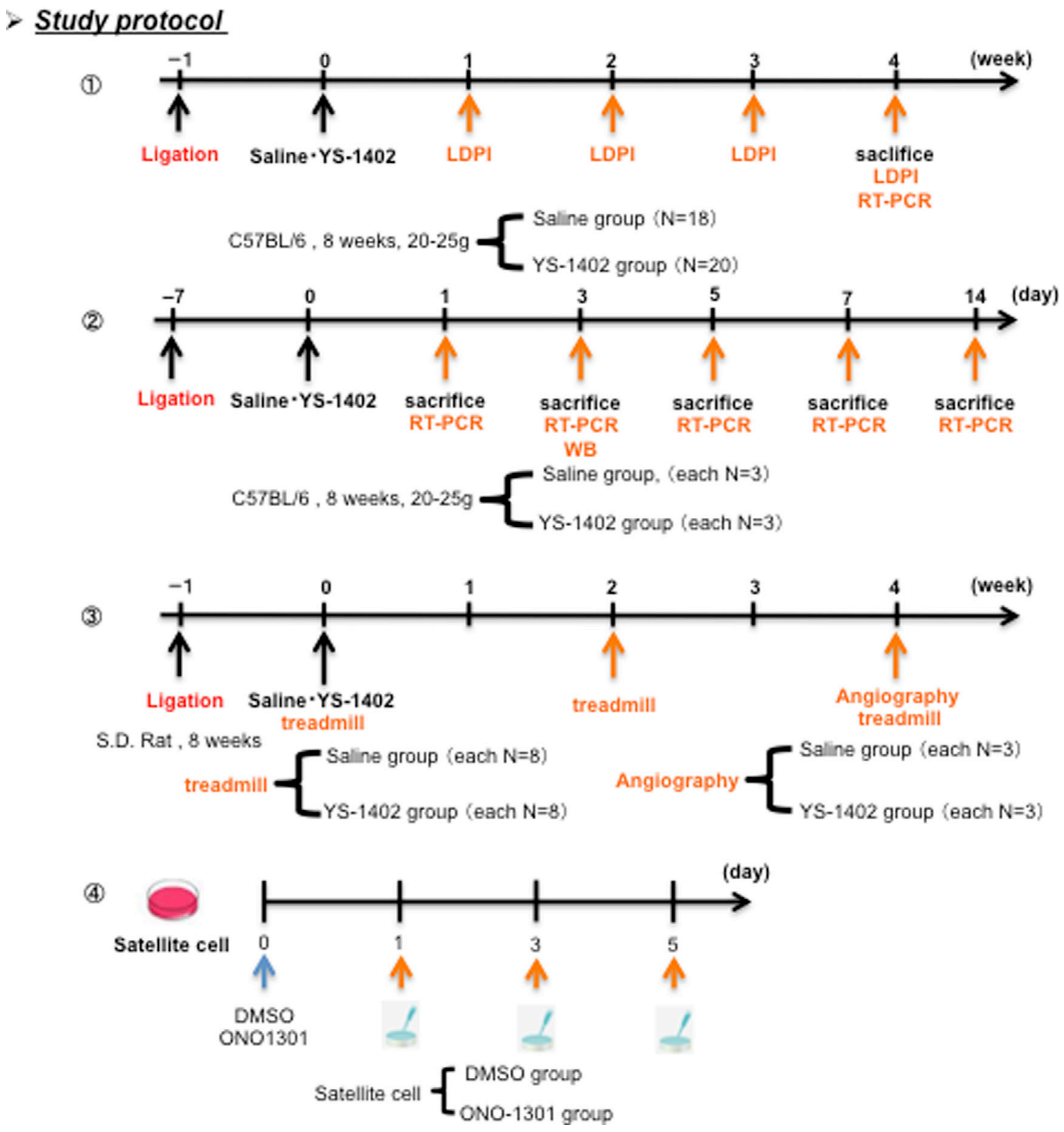

Figure 1. Study Protocol
LDPI, laser Doppler perfusion imaging index; RT-PCR, real-time polymerase chain reaction; WB, western blotting.

S group (group administered saline) (Figure 2A). The LDPI index was significantly higher in the YS group than in the S group 1 week after treatment (71.2% ± 11.4% and 40.1% ± 6.6%, respectively; $p < 0.01$) (Figure 2B).

Increase in Capillary and Arteriolar Density

The YS group demonstrated a significant increase in the number of $CD31^+/\alpha$-smooth muscle actin $(\alpha SMA)^+$ arterioles compared to the S group (458 ± 32.2 and 178 ± 30.6/mm^2, respectively; $p < 0.01$) (Figure 3A).

Gene Expression in the Ischemic Area

For weeks after treatment, the gastrocnemius muscle was obtained to analyze the expression of angiogenesis-related factors (VEGF, HGF, SDF-1), angiogenesis maturation-related factors (angiopoietin-1 [Ang1] and Ang2), bone marrow mesenchymal stem cell induction factor (SDF-1), IPRs, and neural cell adhesion molecule (NCAM) using real-time polymerase chain reaction (PCR). The expression levels of HGF, Ang1, Ang2, SDF-1, IPRs, and NCAM were significantly higher in the YS group than in the S group (S group, 0.56 ± 0.11, 0.98 ± 0.09, 2.86 ± 0.66, 0.68 ± 0.12, 0.67 ± 0.19, 0.87 ± 0.17; YS group,

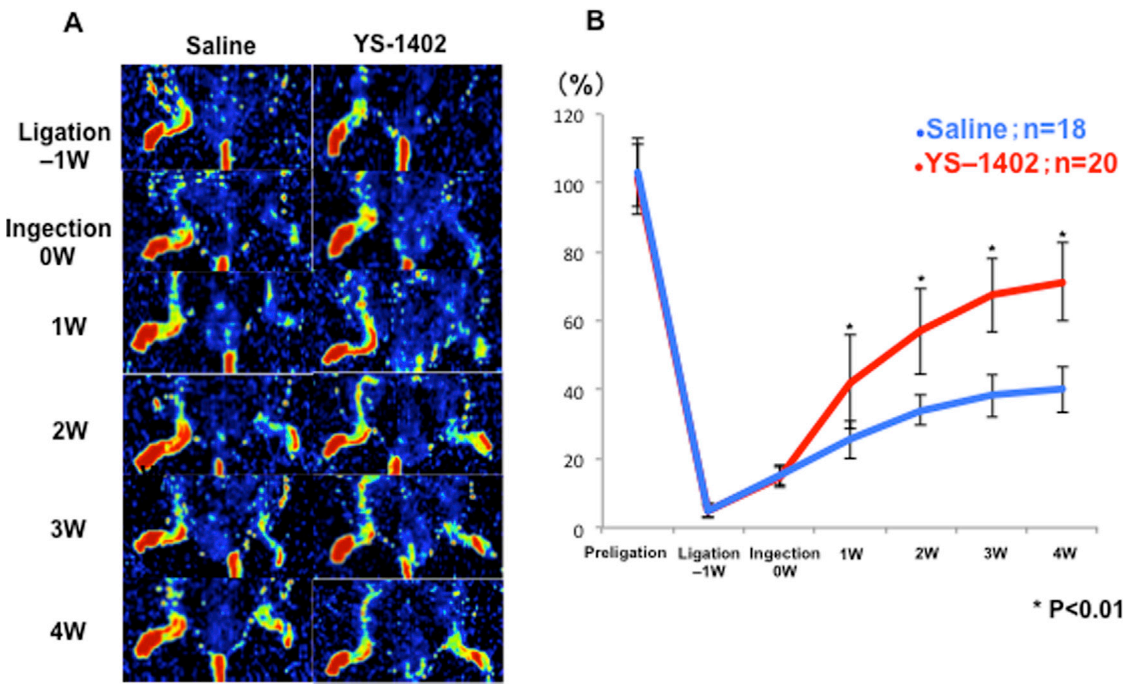

Figure 2. Laser Doppler Perfusion before and after Administration of Saline or YS-1402

(A) Representative laser Doppler perfusion imaging. S group, n = 18; YS group, n = 20. (B) Time course of ischemic/non-ischemic blood perfusion ratio. *$p < 0.01$, **$p < 0.05$ compared with the other group (S group, n = 18; YS group, n = 20).

3.03 ± 0.78, 1.88 ± 0.22, 8.59 ± 1.55, 1.45 ± 0.29, 1.74 ± 0.43, 1.99 ± 0.44, respectively; $p < 0.01$, $p < 0.01$, $p < 0.01$, $p = 0.021$, $p = 0.025$, $p = 0.031$, respectively) (Figure 3B).

Improvement of Exercise Tolerance

The running distance was significantly higher in the YS group than in the S group at 2 and 4 weeks after treatment (2 weeks, 222.5% ± 30.0% and 311.2% ± 61.4%, respectively; $p < 0.01$; 4 weeks, 211.2% ± 73.3% and 295.0% ± 60.3%, respectively; $p = 0.026$) (Figures 4A and 4B).

Angiography in the Ischemic Area

The ratio of the number of blood vessels in the hind leg with lower limb ischemia showed a significant difference between the control and YS-1402 groups (Figures 5A and 5B).

Skeletal Muscle Regeneration

Tissues of the ischemic area were observed by hematoxylin and eosin (H&E) staining at 1, 3, 5, and 7 days after administration. In the YS group, skeletal muscle regeneration was observed from day 3 to day 7 after administration. In contrast, skeletal muscle regeneration was observed beginning on the seventh day after administration in the S group (Figure 6A). In addition, desmin and Ki-67 staining, which reveals skeletal muscle and cell cycle activity, showed double-positive cells around the basement membrane. Furthermore, satellite cell activation was observed (Figure 6B), and thus we stained for the satellite cell markers PAX7 and Ki-67. Their activation site was identical to that in satellite cells, and the number of double-positive cells was significantly higher in the YS group than in the S group (S group, 43.1 ± 25.5/mm^2; YS group, 216.5 ± 29.2/mm^2; $p < 0.01$) (Figures 6C and 6D). Furthermore, to examine the expression of IPRs in satellite cells, we stained for PAX7 and IPRs. The expression of IPRs was higher in the YS group than in the S group (Figure 6E).

Sequential Change of Myoblast-Associated Markers

To determine the time course of skeletal muscle regeneration, changes in the gene expression of skeletal muscle-related factors such as PAX7, MyoD, and myogenin, which are markers of satellite cells, myoblasts, and myofibers, respectively, were examined. Additionally, the ischemic gastrocnemius muscle was examined at 1, 3, 5, 7, and 14 days after treatment. The expression level of PAX7, MyoD, and myogenin was higher in the YS group than in the S group, and the levels of these markers peaked 3 days after YS-1402 administration. In contrast, their levels peaked in the S group 7 days after saline administration (Figure 7A).

Signal Mechanism of Skeletal Muscle Regeneration

To investigate the signaling mechanisms underlying skeletal muscle regeneration, western blotting was performed using protein extracted from a sample collected 3 days after treatment. Western blot analysis revealed that JNK (c-Jun N-terminal kinase), ERK (extracellular signal-regulated kinase), and PI3K

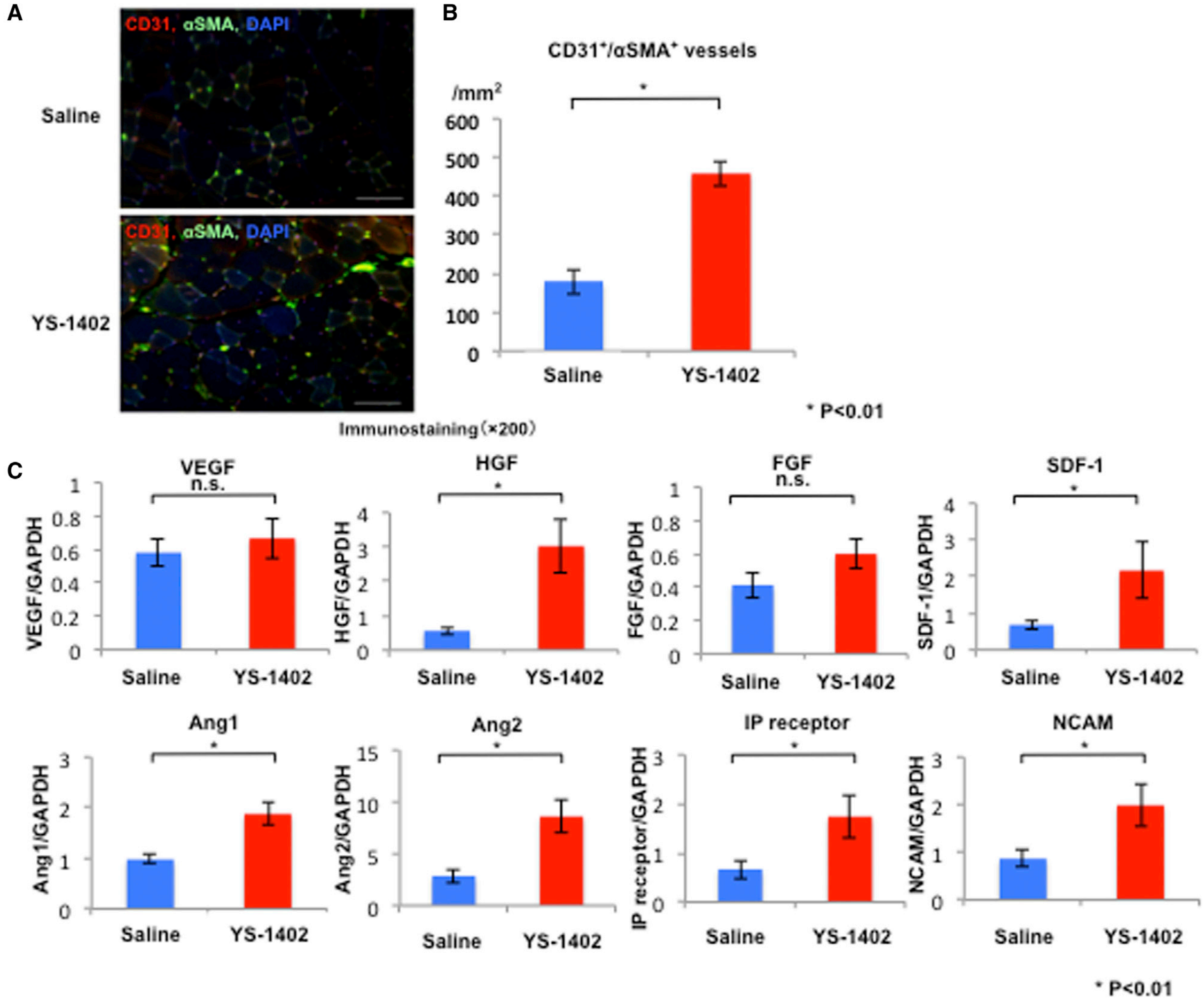

Figure 3. Neovascularization after Administration of YS-1402

(A and B) CD31/αSMA double-positive vessel density. *p < 0.01 compared with the other group (S group, n = 18; YS group, n = 20). Scale bars, 100 μm. (C) Neovascularization-related mRNA, IPR, and NCAM expression in an ischemic area measured by real-time polymerase chain reaction. HGF, SDF-1, Ang1, Ang2, SDF-1, IPR, and NCAM expression levels were significantly higher in the YS group than in the S group. *p < 0.01, **p < 0.05 compared with the other group (S group, n = 18; YS group, n = 20).

(phosphatidylinositol 3-kinase) were associated with skeletal muscle regeneration. No difference was found between JNK and PI3K expression in the S and YS groups (0.65 ± 0.07 versus 1.44 ± 1.21, p = 0.374 and 0.25 ± 0.14 versus 0.45 ± 0.27, p = 0.346, respectively). However, a significant difference in the expression of ERK (S group versus YS group, 0.11 ± 0.01 versus 0.15 ± 0.01; p = 0.025) was found, and it tended to activate pERK (Figure 7B).

Skeletal Muscle Regeneration *In Vitro*

Administration of ONO-1301 increased the number of satellite cells on days 3 and 5, and their differentiation into myoblasts was promoted in the group administered ONO-1301 (Figure 8).

DISCUSSION

This study demonstrated that the administration of YS-1402 improved microcirculation in mice with hindlimb ischemia, increased tissue blood flow, improved limb ischemia, and promoted skeletal muscle regeneration.

In detail, administration of YS-1402 contributed to the increased expression of pro-angiogenic cytokines and IPRs, with YS-1402 acting in the ischemic area. IPRs were increased in the nucleus and cytoplasm of satellite cells, thereby increasing the number of satellite cells. Early after administration, the expression of myoblast-associated markers PAX7, MyoD, and myogenin was confirmed in skeletal muscle.

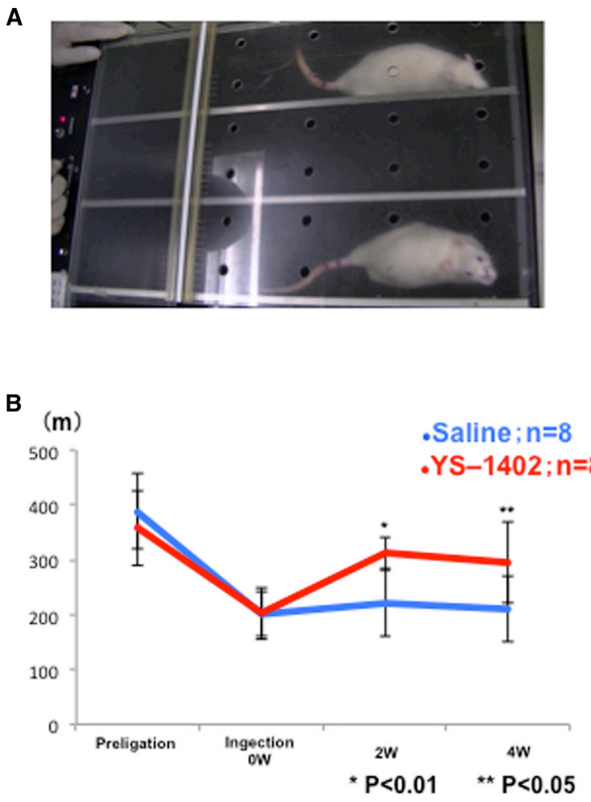

Figure 4. Treadmill Tests

(A) Example of a rat running on the treadmill. (B) Running distance before ligation and at 0, 2, and 4 weeks after YS-1402 administration. *p < 0.01, **p < 0.05 compared with the other group (S group, n = 8; YS group, n = 8).

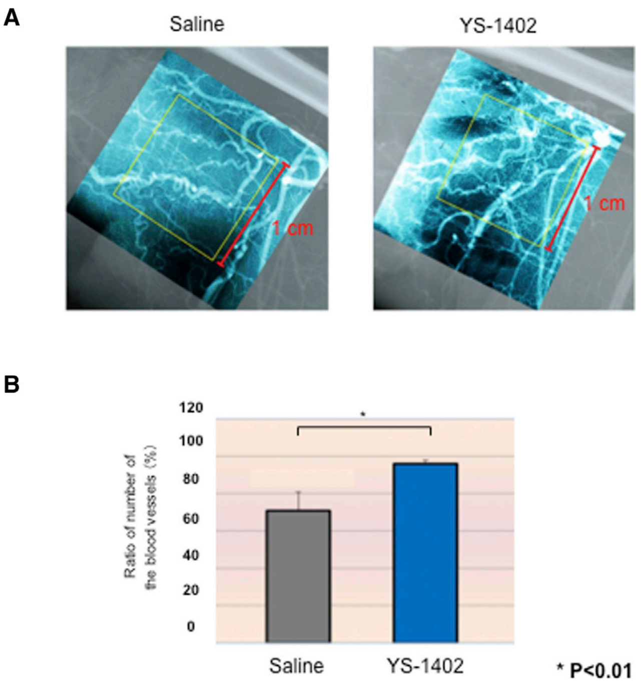

Figure 5. Angiography

(A) Angiography in the ischemic area. (B) Ratio of the number of blood vessels in the hind leg under lower limb ischemia. *p < 0.01, **p < 0.05 compared with the other group (S group, n = 3; YS group n = 3).

YS-1402 administration also resulted in upregulated activity of ERK, a signaling pathway that promotes skeletal muscle regeneration.

YS-1402 was developed as an antiplatelet drug; however, in phase 1 clinical trials, its effective dose induced diarrhea as a side effect.[23,24] Many drug repositioning studies have revealed the clinical effectiveness of YS-1402 at a lower dose without side effects in several heart failure animal models.[22–25] Although most drugs generally exhibit a single therapeutic effect, this anti-heart failure drug has a unique feature, as it exerts multiple therapeutic effects, such as angiogenesis and anti-fibrotic effects, by enhancing the expression of several kinds of cytokines.[17–20,24] These abovementioned mechanisms suggest an idea for regenerative therapy without using cells; that is, this pharmacotherapy may induce regeneration abilities evoked by cells in the living body, with no need for a cell processing center, which is always necessary for cell therapy.

The main pathophysiology of CLI is ischemia of the peripheral limbs resulting from arteriosclerosis and the subsequent breakdown of skeletal muscle owing to ischemia. Thus, to treat this disease, it is important to regenerate the disrupted skeletal muscle simultaneously with angiogenesis.[13]

Previous gene and drug therapies only target angiogenesis, and, therefore, have no effect on degenerating skeletal muscle tissue.[25] However, a prominent feature of YS-1402 is that it can induce angiogenesis with a single dose and can simultaneously induce differentiation and proliferation of satellite cells expressing IPRs. Consequently, the drug is more effective for healing degenerating skeletal muscle.

YS-1402 acts on cells expressing IPRs (such as fibroblasts, smooth muscle cells, and vascular endothelial cells) and induces the secretion of various growth factors, such as VEGF, HGF, and SDF-1.[23] Previous studies have elucidated that VEGF promotes endothelial cell migration and angiogenesis.[26,27] Furthermore, studies have reported that HGF promotes endothelial cell proliferation, angiogenesis, and anti-fibrosis and that this proliferative action is specific to endothelial cells.[28] SDF-1 induces local homing of bone marrow cells through the SDF-1/CXCR4 axis, which promotes formation of neointimal tissue and neovasculogenesis.[29,30] Although single administration of cytokines has been reported to have some difficulties in promoting the maturity of the newly formed vasculature, these three cytokines may be effective potential promotors of functional angiogenesis and may interact with each other to promote angiogenesis.[22,23] Furthermore, these cytokines are considered to act complementarily, leading to effective vascular tissue regeneration.

Basic research on YS-1402 for various heart failure models reports that this preparation is effective for ischemic cardiomyopathy

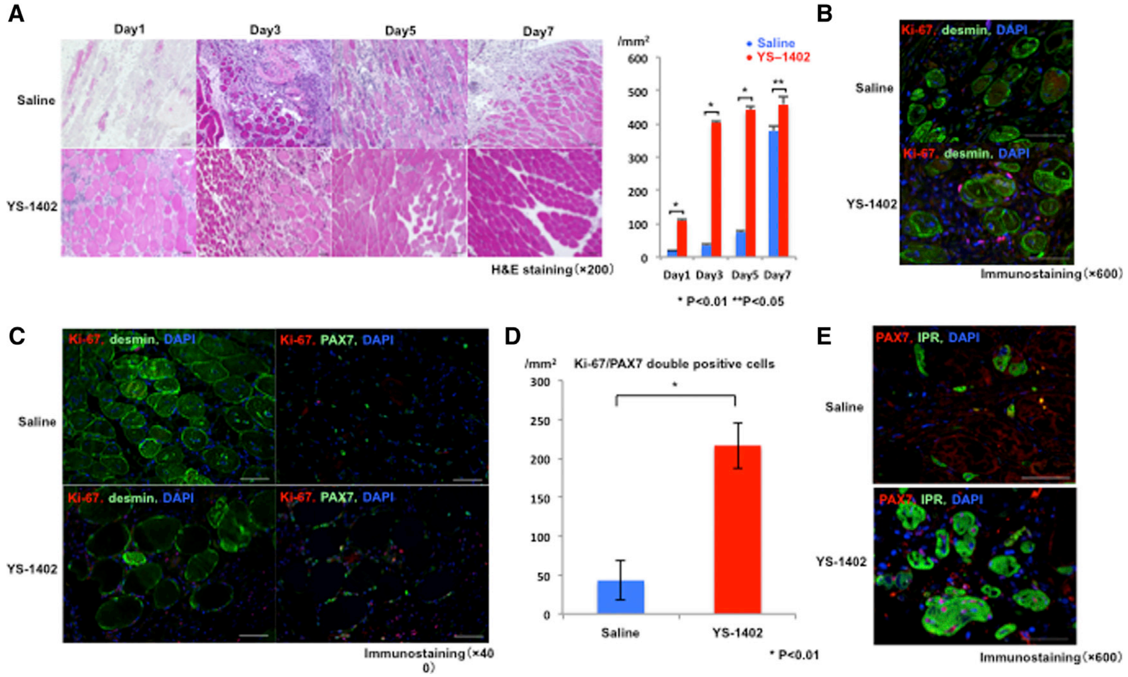

Figure 6. Skeletal Muscle Regeneration after Administration of YS-1402

(A) H&E staining of the ischemic area at 1, 3, 5, and 7 days after administration of saline or YS-1402. *p < 0.01 compared with the other group (n = 3 for each group). (B) Desmin and Ki-67 staining, which shows skeletal muscle and cell cycle activity. (C) PAX7 and Ki-67 staining for satellite cell activity. (D) PAX7/Ki-67 double-positive cells. *p < 0.01 compared with the other group (n = 3 for each group). (E) Expression of prostacyclin receptor in satellite cells. Scale bars, 50 μm.

models and dilated cardiomyopathy models. The mechanism is thought to be a functional angiogenic effect via multiple cytokines. This mechanism resulted in improved microcirculation in mice with hindlimb ischemia, increased tissue blood flow, and improved limb ischemia as well as exercise durability (Figure 8).

When a skeletal muscle is damaged, the signal transduction pathways of skeletal muscle regeneration, such as JNK, ERK, and PI3K, are activated, resulting in the activation of muscle satellite cells, their differentiation into myoblasts and myofibers, and muscle regeneration.[31] Enhancement of this process may play a crucial role in tissue regeneration in CLI. Nishida et al.[32] reported that PGE2 promotes the differentiation of myocytes by promoting myotube formation. In this study, the expression of PAX7, MyoD, and myogenin was high in the YS-1402 group, and ERK activity was upregulated. This indicates that YS-1402 may have promoted the differentiation of satellite cells to skeletal muscle. An experiment that administered ONO-1301 *in vitro* confirmed that the differentiation of satellite cells into myoblasts was promoted in the group administered ONO-1301. In this study, whether this drug induced HGF or directly acted on myoblasts to regenerate damaged skeletal tissue is a major theme.

Tatsumi et al.[33] reported that HGF acts on the c-Met gene, which activates both signal transduction pathways of skeletal muscle regeneration, such as ERK, and satellite cells. Therefore, in this study, YS-1402 acted on fibroblasts, vascular smooth muscle cells, and vascular endothelial cells, and the continuously released HGF acted on c-Met to activate satellite cells, thereby possibly promoting muscle regeneration. This indirect pathway may also promote skeletal muscle regeneration[33,34] (Figure 9).

Although YS-1402 has a potential to exert direct action on satellite cells, no studies concerning its direct action have been reported. However, one member of the prostaglandin family, PGF2a, is synthesized when Ca^{2+} flows into cells due to skeletal muscle damage and when cPLA2 is activated and arachidonic acid is metabolized in the cytoplasm. The synthesized PGF2a is then released outside the cell. It has been reported that PGF2a released to the extracellular space binds to IPR and activates P13K, JNK, and ERK to induce their transcription and regeneration and enhancement of damaged muscle.[31] In this study, YS-1402 administration upregulated ERK activity and promoted myoblast differentiation. Therefore, similar to PGF2a, it is also possible that YS-1402 directly bound to IPR, activated ERK, and promoted myoblast differentiation. Thus, both the direct action of YS-1402 and its indirect action via HGF are thought to be involved in the mechanism underlying its role in skeletal muscle regeneration. Future studies are warranted to elucidate the mechanisms associated with skeletal muscle regeneration.

In conclusion, YS-1402 administration promoted functional angiogenesis and skeletal muscle regeneration and increased

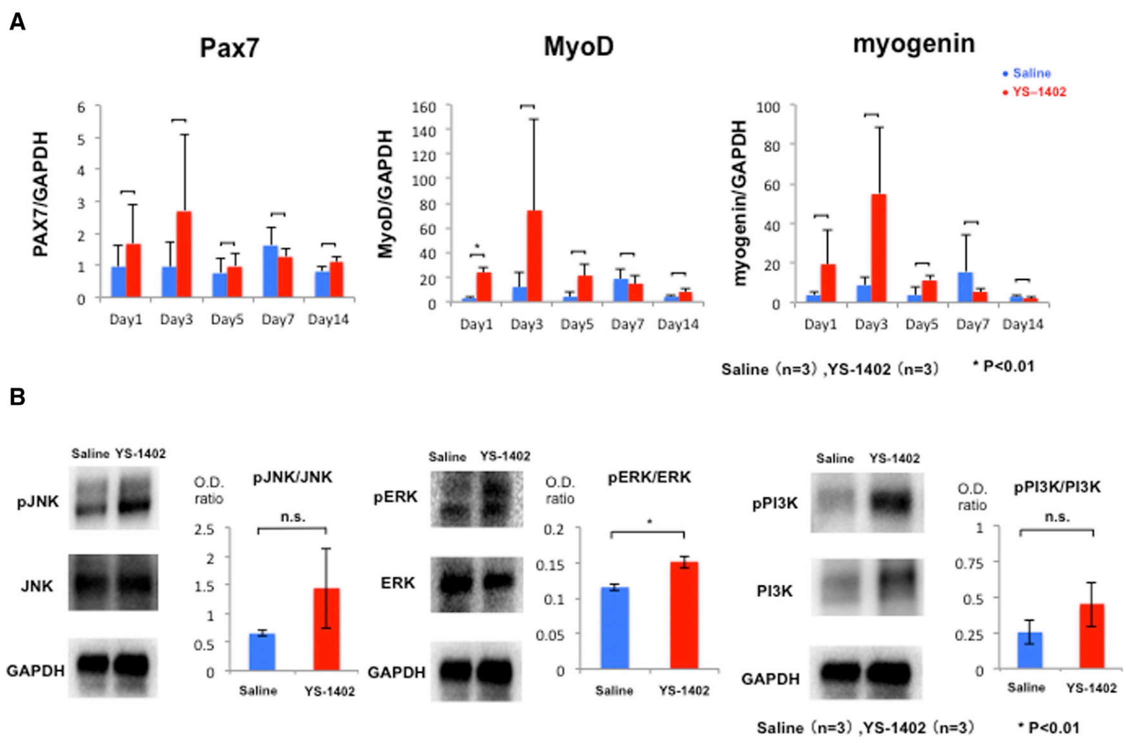

Figure 7. Skeletal Muscle Regeneration-Related Factor

(A) Skeletal muscle regeneration-related mRNA expression in an ischemic area at 1, 3, 5, and 7 days after treatment measured by real-time PCR. *p < 0.01, **p < 0.05 compared with the other group (n = 3 for each group). (B) Skeletal muscle regeneration-related protein levels in an ischemic area at 3 days after treatment measured by western blot analysis. *p < 0.01, **p < 0.05 compared with the other group (n = 3 for each group).

blood flow in a CLI mouse model, suggesting a new therapeutic strategy for arteriosclerosis obliterans (ASO).

MATERIALS AND METHODS

Animals and Ethical Considerations

For this study, 4- or 8-week-old male C57BL/6J wild-type mice were purchased from CLEA Japan (Osaka, Japan) and 8-week-old male WI rats were purchased from Charles River Laboratories Japan (Yokohama, Japan). All animal protocols were approved by the Animal Experimentation Committee of Osaka University. All animal experiments were performed according to the Guidelines for Animal Experiments of Osaka University.

Animal care was reviewed and approved based on the *Guide for the Care and Use of Laboratory Animals*. We took care of the animals in a controlled, non-stressed environment for at least 1 week before the beginning of the study to avoid possible confounding variables related to constructive preconditioning.

Preparation of YS-1402

YS-1402 (Ono Pharmaceutical, Osaka, Japan) contains polylactic acid and glycolic acid at a ratio of 50:50 and is the polymerized form of ONO-1301 because it is designed to achieve a sustained-release system. YS-1402 was polymerized with PLGA microspheres as follows: ONO-1301 and PLGA (50:50 ratio of polylactic acid and glycolic acid) were dissolved in dichloromethane. The dissolved polymer was added to an aqueous solution of polyvinyl alcohol to form an oil-in-water emulsion. Then, the emulsion was stirred at room temperature to allow evaporation of dichloromethane. After centrifugation and washing, YS-1402 was isolated by lyophilization.

Preparation Method of YS-1402 Medium 40-mL Solution

First, 2.5 g of mannitol was weighed, dissolved in water for injection, and made up to 50 mL to prepare a 5% (w/v) solution. Then, 80 mg of polysorbate 80 was weighted and put it in a graduated cylinder containing an appropriate amount of 5% (w/v) mannitol solution, after which another 5% (w/v) mannitol solution was made up to 40 mL, and mixed by inverting. The 5% (w/v) mannitol solution containing 0.2% (w/v) polysorbate 80 (hereinafter, medium) was filtered in a clean bench using a 0.220-μm membrane filter and a polypropylene disposable syringe. The prepared medium (filtrate) was then put in a sterilized brown polypropylene container and stored in a refrigerator (4°C).

Preparation Method of YS-1402 Administration Solution

For YS-1402, 100 mg was precisely weighed and suspended in 1.6 mL of medium (10 mg/mL solution was prepared as ONO-1301). For the preparation of YS-1402, the medium was added after weighing, and

the mixture was vortexed. In addition, sterilization equipment was used for the preparation, and it was performed aseptically in a safety cabinet. After the preparation, it was stored on ice until just before administration.

Animal Preparation and Experimental Protocol

Prior to surgery and LDPI, the mice and rats were anesthetized with a combination anesthetic (M/M/B: 0.3/4/5). A dose of 5 mL/kg was administered via intraperitoneal injection. A skin incision was made in the left hindlimb. The proximal femoral artery and proximal portion of the saphenous artery were ligated. After all side arterial branches were dissected, the entire femoral artery was excised.

One week after surgery, blood flow ratio was measured LDPI, and ischemia (10%–20% ischemia) model animals were selected and randomly divided into two groups, that is, the S group and the YS group. We injected 0.03 mL of saline or YS-1402 into the ischemic thigh adductors immediately after induction of ischemia. The injections were performed according to the following protocol.

Protocol 1

Blood flow ratio was measured by LDPI before ligation, after ligation, before administration, after administration, and 1, 2, 3, and 4 weeks after surgery. The gastrocnemius muscle was dissected 4 weeks after administration of saline or YS-1402 and tissue staining and real-time PCR were performed (n = 18 for S group, n = 20 for YS group) (Figure 1).

Protocol 2

The gastrocnemius muscle tissue was collected 1, 3, 5, 7, and 14 days after administration, and tissue staining and real-time PCR were performed. Western blots were performed on day 3 samples (n = 3 for each group) (Figure 1).

Protocol 3

Using 8-week-old WI rats, a treadmill test was performed before ligation and 0, 2, and 4 weeks after administration, and the running distance was measured. Furthermore, angiography was performed 4 weeks after administration (n = 8 for each group) (Figure 1).

Protocol 4

Satellite cells were isolated from 4-week-old mice and treated with DMSO or ONO-1301. After treatment, satellite cells were collected 1, 3, and 5 days later and then immunostained (Figure 1).

LDPI Analysis

Hindlimb blood perfusion was scanned using an LDP image analyzer (PeriScan PIM III, Perimed, Järfälla, Sweden). Ischemic and non-ischemic limb perfusion was measured before ligation, after ligation, before administration, after administration, and at 1, 2, 3, and 4 weeks after surgery. The ratio of ischemic to non-ischemic hindlimb perfusion was calculated. To obtain consistent results and reduce the confounding effects of temperature, we fully warmed the mice using a heating pad for 5 min before analyses.

Histological Analyses

The gastrocnemius tissues were formalin fixed and paraffin embedded and cut into 5-μm sections using a microtome for histological analyses. The sections were stained by H&E and periodic acid-Schiff (PAS) staining. The images were examined using an optical microscope (Keyence, Osaka, Japan), and quantitative morphometric analysis was performed for each sample using Metamorph software (Molecular Devices, Sunnyvale, CA, USA). Using immunohistochemistry methods, the sections were labeled with polyclonal anti-antibody (anti-CD31; Abcam, ab28364) and were visualized by the LSAB kit (Dako, Glostrup, Denmark, K0690), which is an automated immunostaining system based on the Lepto-streptavidin-biotin-peroxidase method. Again, using immunohistochemistry, the sections were labeled with antibodies (SMA, Dako, M0851; Ki-67, Abcam, ab16667; myogenin, Abcam, ab1835; MyoD, Abcam, ab16148; PAX7, Abcam, ab199010; IPR, Abcam, ab60706), visualized using the corresponding secondary antibodies (Alexa Fluor 488 or Alexa Fluor 555, Molecular Probes, Eugene, OR, USA) that were counterstained by a Hoechst 33342 solution (Dojindo, Kumamoto, Japan, EJ-091), and assessed using a confocal microscope (Olympus, Tokyo, Japan).

RNA Extraction

Total RNA was isolated from the gastrocnemius samples using the RNeasy kit (QIAGEN, Hilden, Germany) as follows. The collected gastrocnemius muscle, 300 μL of buffer RLT, and 3 μL of 2-mercaptoethanol were placed in a tube and disrupted with a tissue lyser (20 Hz, 2 min). The lysate was then centrifuged for 3 min at maximum speed. Thereafter, the supernatant was carefully removed by pipetting, transferred to a guide RNA (gDNA) eliminator spin column placed in a 2-mL collection tube, and then 590 μL of RNase-free water and 10 μL of proteinase K were added. Next, the mixture was centrifuged for 3 min at 20°C–25°C and 10,000 × *g*. The sample solution was then transferred into a new 1.5-mL tube and then 450 μL of ethanol (96%–100%) was added.

Thereafter, 700 μL of the sample solution was collected and applied to an RNeasy mini spin column set in a 2-mL collection tube and then centrifuged at 20°C–25°C and 8,000 × *g* for 1 min. The filtrate was discarded. The same procedure was performed for the remaining sample solutions. Next, 350 μL of buffer RW1 was added to the RNeasy spin column and then centrifugation was carried out at 20°C–25°C and 8,000 × *g* for 1 min, and then the filtrate was discarded. Thereafter, 10 μL of DNase I solution was added to 70 μL of buffer RDD and centrifuged. This DNase I incubation solution was directly applied to the RNeasy spin column with a pipette, and then incubation was carried out for 15 min at room temperature. Next, 350 μL of buffer RW1 was added to the RNeasy spin column and then centrifugation was performed at 20°C–25°C and 8,000 × *g* for 1 min, and the filtrate was discarded. Thereafter, 500 μL of buffer RPE was added to the RNeasy spin column and then centrifugation was conducted at 20°C–25°C and 8,000 × *g* for 1 min, and the filtrate was discarded. Next, 500 μL of buffer RPE was added to the RNeasy spin column and then centrifugation was carried out at

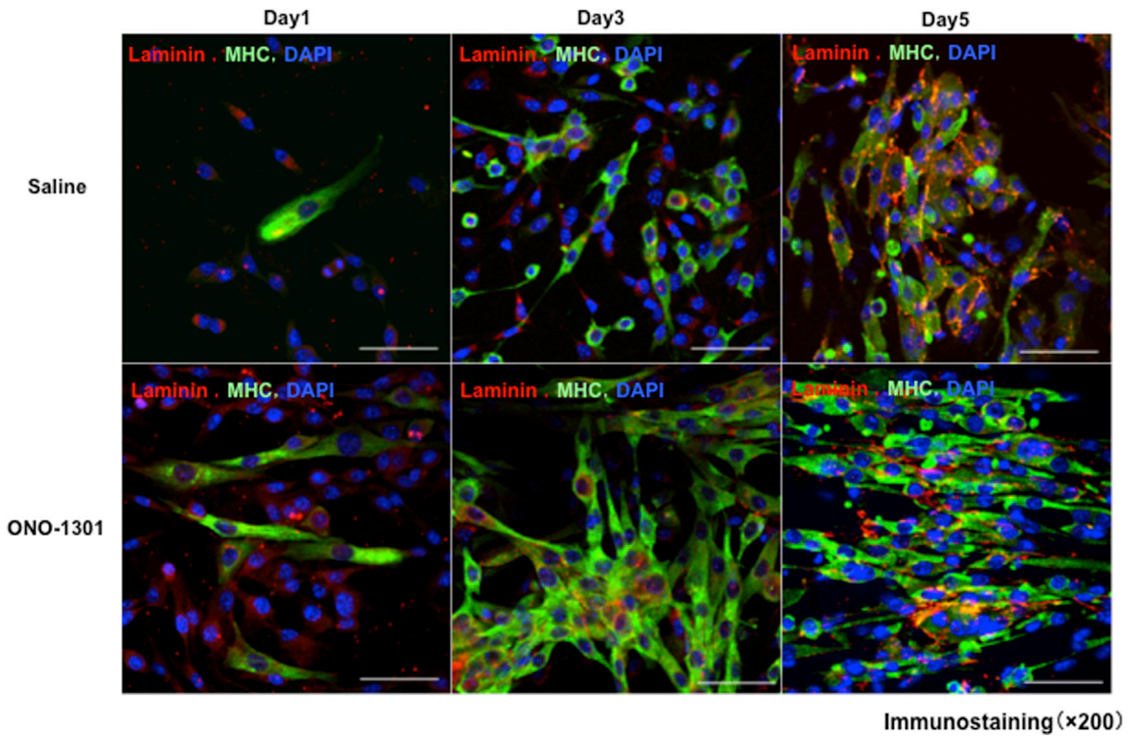

Figure 8. Skeletal Muscle Regeneration after ONO1301 Administration *In Vitro*

MHC (major histocompatibility complex) and laminin staining for satellite cells. Administration of ONO-1301 increased the number of satellite cells on days 3 and 5.

20°C–25°C and 8,000 × *g* for 2 min, and the filtrate was discarded. The sample was then transferred to a new RNeasy spin column collection tube and centrifuged at 20,000 × *g* for 1 min. Next, a 1.5-mL collection tube was placed on the RNeasy spin column, and then 30 μL of RNase-free water was added to the RNeasy spin column followed by centrifugation at 20°C–25°C and 8,000 × *g* for 1 min to extract RNA.

Real-Time PCR

The total RNA was reverse transcribed using the Omniscript reverse transcriptase kit (QIAGEN). Real-time PCR was performed using the TaqMan gene expression assay master mix (Applied Biosystems, Foster City, CA, USA) on the 7500 Fast real-time PCR system (Applied Biosystems, Foster City, CA, USA).

Expression of each mRNA was normalized to that of glyceraldehyde-3-phosphate dehydrogenase (GAPDH).

The following genes were analyzed using the TaqMan gene expression assay (Applied Biosystems): VEGF (Mm01281449_m1), HGF (Mm01135184_m1), fibroblast growth factor (Mm01285715_m1), SDF-1 (Mm00445553_m1), platelet-derived growth factor receptor-α (Mm00456503_m1), NCAM (Ss03391855_m1), prostaglandin I receptor (Mm00801939_m1), Ang1 (Mm00833184_s1), Ang2 (Mm00657574_m1), PAX7 (Mm01354484_m1), MyoD (Mm00521984_m1), and myogenin (Mm00446194_m1). GAPDH (Mm99999915_g1) was co-amplified as an internal control for RNA integrity.

Treadmill

The rats ran on a treadmill at an angle of 15° and speed of 15 m/min. The speed was stepped up by 5 m/min at 5-min intervals. The rats ran on the treadmill, until they could not run, and the running distance was measured.

Angiography

The rats were fixed in the dorsal position under 2% inhalation anesthesia with isoflurane (lot no. 139ANH, Mylan Pharmaceutical), a cannula connected to a pressure transducer was inserted into the carotid artery, and blood pressure and heart rate were measured. After the measurement, the animals were exsanguinated by incising the carotid artery. After exsanguination, the upper part of the abdominal aorta was ligated, and microfilament (MV-122, yellow), a contrast medium, was injected into the abdominal aorta to sacrifice the left and right hindlimbs. X-ray images were taken using Softex (soft X-ray equipment). Image analysis was performed to count the number of blood vessels crossing the 1-cm^2 grid (0.5-mm^2 grid) at the normal site and the ischemic site. The ratio (%) of the number of blood vessels in the ischemic area to that in the normal area was calculated. The microvessel diameter that can be measured by this measurement method was approximately 100–200 μm.

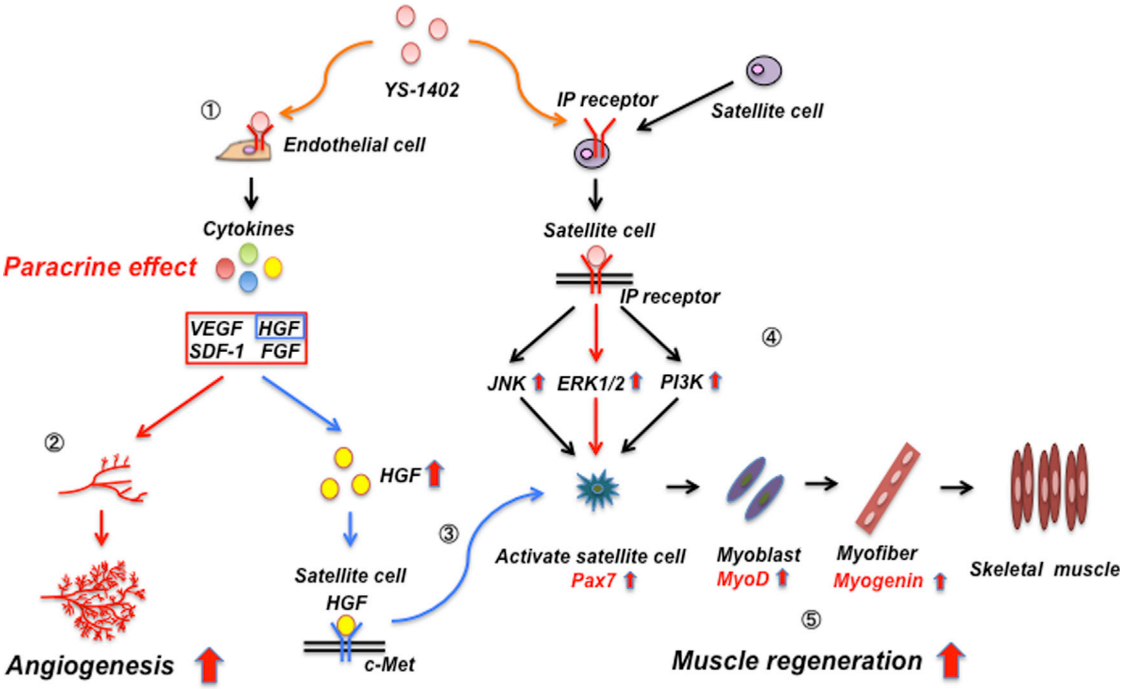

Figure 9. Mechanism of Angiogenesis and Skeletal Muscle Regeneration after Administration of YS-1402
1→2: Mechanism of angiogenesis. 1→3→5: Indirect action of YS-1402 on skeletal muscle regeneration. 4→5: Direct action of YS-1402 on satellite cells.

Protein Quantitation

Protein concentration was quantified using a protease inhibitor (Santa Cruz Biotechnology) and radioimmunoprecipitation assay (RIPA) lysis buffer (Santa Cruz Biotechnology). The Takara BCA (bicinchoninic acid) protein assay kit (Takara Bio) was used as follows.

A solution was prepared by dissolving the VEGI standard with Calidra TOR diluent. The collected gastrocnemius muscle was placed in this solution and centrifuged (400 × g, 4°C, 5 min). This was used as the sample solution. A 1.5-mL tube, a 96-well plate, and a 10-fold diluted sample solution were prepared. A standard solution was then prepared in a 1.5-mL tube (25 μL) at various concentrations (2, 1, 0.5, 0.25, 0.125, 0.0625, and 0 mg/mL). The sample solution (25 μL) was poured into a 1.5-mL tube, and then solution A and solution B were mixed to prepare a coloring solution (A/B ratio of 50:1). The coloring solution (200 μL) was added to the tubes containing the standard solution and the sample. The standard solution and the sample were then added into wells in a 96-well plate and incubated at 37°C for 30 min. The absorbance was measured with an absorptiometer.

Western Blotting

For western blotting, 10 mg of protein was electrophoresed with 4%–12% PROTEAN precast gels (Bio-Rad) and transferred to a polyvinylidene fluoride blotting membrane (GE Healthcare Life Science). The membrane was incubated with the primary antibody overnight at 4°C, followed by incubation with the secondary antibody conjugated with horseradish peroxidase (1:2,000). Signals were detected and visualized using Amersham ECL (enhanced chemiluminescence) Prime western blotting detection reagent (GE Healthcare). Luminescence was detected using a ChemiDoc MP imaging system (Bio-Rad).

Quantification of the band intensity was performed with Image Lab software version 5.0 (Bio-Rad). Day 3 samples were analyzed. The following primary antibodies were used: anti-phosphorylated-JNK, anti-JNK (1:1,000; Abcam); anti-phosphorylated-ERK, anti-ERK (1:1,000; Cell Signaling Technology); anti-phosphorylated-PI3K, anti-PI3K (1:1,000; Abcam); and anti-GAPDH (1:1,000; Abcam). Amersham ECL anti-rabbit immunoglobulin G (IgG) (GE Healthcare) and Amersham ECL anti-mouse IgG (GE Healthcare) were used as secondary antibodies, each at a dilution of 1:2,000.

Isolation of Satellite Cells

Isolation of Mononuclear Cells from Mouse Skeletal Muscle

One or two adult mice (4 weeks) were sacrificed, and then the skin was pinched and slit off the abdomen using sharp scissors. Thereafter, the skin was completely peeled off to reveal the triceps and hindlimb muscles. Next, all leg skeletal muscles (tibialis anterior, gastrocnemius, and quadriceps) and triceps along the bones were removed using scissors, after which the muscles were transferred into ice-cold, sterile phosphate-buffered saline (PBS) in a 10-cm plate. Next, the muscles were cleaned of blood using PBS and transferred to a new 6-cm plate (one plate for one to two mice). Thereafter, connective tissue, blood vessels, nerve bundles, and adipogenic tissue were

removed under a dissection microscope, and then the tissues were cut and minced into a smooth pulp. The minced muscles were then transferred to 50-mL Falcon tubes and then 5 mL of collagenase solution (0.2% collagenase type 2 in 10% fetal bovine serum [FBS] in Dulbecco's modified Eagle's medium [DMEM]) was added, followed by incubation at 37°C for 60 min. The mixture was then homogenized by trituration (up and down with an 18G needle) and further incubated at 37°C for 15 min, followed by another round of trituration to dissociate the mixture into a single suspension, and then up to 50 mL of 2% DMEM was added and mixed well. Next, a cell strainer (70 μm) was placed onto the Falcon tube and then the supernatant containing the dissociated cells was transferred onto the cell strainer. The cell suspension was then pipetted up and down until it passed through the strainer. Thereafter, the number of cells was counted using a hemocytometer, and then the tubes were centrifuged at 2,000 rpm at 4°C for 5 min, after which the supernatant was aspirated and discarded. The mixture was then resuspended with 200 μL of 2% FBS in DMEM and transferred into 1.5-mL microcentrifuge tubes. Approximately 2×10^6 cells were harvested from muscles of one mouse. The cells were diluted to a concentration of 1×10^6 cells in 100 μL of 2% FBS in DMEM.

Antibody Staining and Separation with Magnetic-Activated Cell Sorting (MACS)

During the following procedures, sterile conditions were maintained using sterile buffers, and each volume of the added antibodies and cell suspension medium was calculated for cells from whole muscles of one mouse (in cases where cells are harvested from two or more mice, the amount of reagents should be optimized). One microliter each of CD31-phycoerythrin (PE), CD45-PE, Sca-1-PE, and integrin α7 antibody was added to 200 μL of cell suspension and incubated on ice for 30 min. Thereafter, 1 mL of 2% FBS in DMEM was added to the cell suspension in a 1.5-mL tube and centrifuged at 2,000 rpm at 4°C for 3 min. This step was repeated twice. Next, the supernatant was aspirated and discarded. The cells were then resuspended with 200 μL of 2% FBS in DMEM, followed by the addition of 10 μL of anti-PE magnetic beads and incubation on ice for 30 min. Next, 1 mL of MACS buffer was added to the cell suspension in a 1.5-mL microcentrifuge tube and then centrifuged at 2,000 rpm at 4°C for 3 min. This step was repeated twice. The supernatant was then aspirated and discarded. Thereafter, the cells were resuspended with 1.0 mL of MACS buffer. An LD column was sept up on a magnetic board and rinsed with 2.0 mL of MACS buffer. The cell suspension was then transferred onto the LD column and the flowthrough fraction was collected into a 1.5-mL tube. This fraction contained PE-negative cells. The tube was then centrifuged at 2,000 rpm at 4°C for 3 min, after which the supernatant was aspirated and discarded. Next, the cells were resuspended with 200 μL of 2% FBS in DMEM, followed by the addition of 10 μL of anti-mouse IgG magnetic beads and incubation on ice for 30 min. Next, 1 mL of MACS buffer was added to the cell suspension in a 1.5-mL tube and then centrifuged at 2,000 rpm at 4°C for 3 min, and then the supernatant was aspirated and discarded. This step was repeated twice, and then the cells were resuspended with 500 μL of MACS buffer.

An MS column was sept up on a magnetic board and rinsed with 500 μL of MACS buffer. The suspended cell solution was then transferred onto the column and then the flowthrough fraction (integrin α7-negative cells) was discarded. The column was rinsed with 1 mL of MACS buffer and then the above step was repeated twice. After rinsing, the column was removed from the magnetic field of the separator, and then 1 mL of MACS buffer was applied onto the column to elute the magnetically labeled cells (integrin α7-positive cells) into a 1.5-mL microcentrifuge tube by pushing the syringe plunger from the top of the column. The flowthrough fraction was then collected into a 1.5-mL tube. The elution was repeated with 1.5 mL of MACS buffer and then the flowthrough fraction was collected. The collected fraction was centrifuged at 2,000 rpm at 4°C for 3 min, and then the supernatant was aspirated and discarded. The purified cells were then resuspended with 1 mL of myoblast medium (20% FBS containing Ham's F-10 with basic fibroblast growth factor [bFGF]) and plated on a Matrigel-coated 10-cm plate with 8 mL of myoblast medium (5 mL in a 6-cm plate).

Statistical Analyses

Data were analyzed using JMP Pro 13 (SAS Institute, Cary, NC, USA). All data except PCR results are presented as mean ± standard deviation. PCR results are presented as mean ± standard error. Differences between groups were determined using the Student's t test. Differences were considered statistically significant when $p < 0.05$.

SUPPLEMENTAL INFORMATION

Supplemental Information can be found online at https://doi.org/10.1016/j.omtm.2020.05.022.

AUTHOR CONTRIBUTIONS

T.N., S.M., and Y.S. proposed experimental ideas. T.N., S.M., and A.H. designed the experiments and conducted data processing and statistical analyses. T.N., S.M., and Y.S. wrote the manuscript and made critical revisions/analyses. All authors approved the final copy.

CONFLICTS OF INTEREST

The authors declare no competing interests.

ACKNOWLEDGMENTS

We thank Soichiro Fukada for excellent technical assistance.

REFERENCES

1. Ouriel, K. (2001). Peripheral arterial disease. Lancet *358*, 1257–1264.
2. Norgren, L., Hiatt, W.R., Dormandy, J.A., Nehler, M.R., Harris, K.A., and Fowkes, F.G.; TASC II Working Group (2007). Inter-society consensus for the management of peripheral arterial disease. J. Vasc. Surg *45* (*Suppl S*), S5–S67.
3. Teraa, M., Conte, M.S., Moll, F.L., and Verhaar, M.C. (2016). Critical limb ischemia: current trends and future directions. J. Am. Heart Assoc. *5*, e002938.
4. Tu, C., Das, S., Baker, A.B., Zoldan, J., and Suggs, L.J. (2015). Nanoscale strategies: treatment for peripheral vascular disease and critical limb ischemia. ACS Nano *9*, 3436–3452.
5. Baumgartner, I., Pieczek, A., Manor, O., Blair, R., Kearney, M., Walsh, K., and Isner, J.M. (1998). Constitutive expression of $phVEGF_{165}$ after intramuscular gene transfer

promotes collateral vessel development in patients with critical limb ischemia. Circulation *97*, 1114–1123.

6. Ruel, M., Al-Faleh, H., Kulik, A., Chan, K.L., Mesana, T.G., and Burwash, I.G. (2006). Prosthesis-patient mismatch after aortic valve replacement predominantly affects patients with preexisting left ventricular dysfunction: effect on survival, freedom from heart failure, and left ventricular mass regression. J. Thorac. Cardiovasc. Surg. *131*, 1036–1044.

7. Lederman, R.J., Mendelsohn, F.O., Anderson, R.D., Saucedo, J.F., Tenaglia, A.N., Hermiller, J.B., Hillegass, W.B., Rocha-Singh, K., Moon, T.E., Whitehouse, M.J., and Annex, B.H.; TRAFFIC Investigators (2002). Therapeutic angiogenesis with recombinant fibroblast growth factor-2 for intermittent claudication (the TRAFFIC study): a randomised trial. Lancet *359*, 2053–2058.

8. Ferrara, N., and Alitalo, K. (1999). Clinical applications of angiogenic growth factors and their inhibitors. Nat. Med. *5*, 1359–1364.

9. Henry, T.D., Annex, B.H., McKendall, G.R., Azrin, M.A., Lopez, J.J., Giordano, F.J., Shah, P.K., Willerson, J.T., Benza, R.L., Berman, D.S., et al.; VIVA Investigators (2003). The VIVA trial: vascular endothelial growth factor in ischemia for vascular angiogenesis. Circulation *107*, 1359–1365.

10. Cobellis, G., Silvestroni, A., Lillo, S., Sica, G., Botti, C., Maione, C., Schiavone, V., Rocco, S., Brando, G., and Sica, V. (2008). Long-term effects of repeated autologous transplantation of bone marrow cells in patients affected by peripheral arterial disease. Bone Marrow Transplant. *42*, 667–672.

11. Ruiz-Salmeron, R., de la Cuesta-Diaz, A., Constantino-Bermejo, M., Pérez-Camacho, I., Marcos-Sánchez, F., Hmadcha, A., and Soria, B. (2011). Angiographic demonstration of neoangiogenesis after intra-arterial infusion of autologous bone marrow mononuclear cells in diabetic patients with critical limb ischemia. Cell Transplant. *20*, 1629–1639.

12. Franz, R.W., Parks, A., Shah, K.J., Hankins, T., Hartman, J.F., and Wright, M.L. (2009). Use of autologous bone marrow mononuclear cell implantation therapy as a limb salvage procedure in patients with severe peripheral arterial disease. J. Vasc. Surg *50*, 1378–1390.

13. Wang, X., Zhang, J., Cui, W., Fang, Y., Li, L., Ji, S., Mao, D., Ke, T., Yao, X., Ding, D., et al. (2018). Composite hydrogel modified by IGF-1C domain improves stem cell therapy for limb ischemia. ACS Appl. Mater. Interfaces *10*, 4481–4493.

14. Nakamura, K., Sata, M., Iwata, H., Sakai, Y., Hirata, Y., Kugiyama, K., and Nagai, R. (2007). A synthetic small molecule, ONO-1301, enhances endogenous growth factor expression and augments angiogenesis in the ischaemic heart. Clin. Sci. (Lond.) *112*, 607–616.

15. Hirata, Y., Soeki, T., Akaike, M., Sakai, Y., Igarashi, T., and Sata, M. (2009). Synthetic prostacycline agonist, ONO-1301, ameliorates left ventricular dysfunction and cardiac fibrosis in cardiomyopathic hamsters. Biomed. Pharmacother. *63*, 781–786.

16. Hirata, Y., Kurobe, H., Uematsu, E., Yagi, S., Soeki, T., Yamada, H., Fukuda, D., Shimabukuro, M., Nakayama, M., Matsumoto, K., et al. (2013). Beneficial effect of a synthetic prostacyclin agonist, ONO-1301, in rat autoimmune myocarditis model. Eur. J. Pharmacol. *699*, 81–87.

17. Imanishi, Y., Miyagawa, S., Fukushima, S., Ishimaru, K., Sougawa, N., Saito, A., Sakai, Y., and Sawa, Y. (2013). Sustained-release delivery of prostacyclin analogue enhances bone marrow-cell recruitment and yields functional benefits for acute myocardial infarction in mice. PLoS ONE *8*, e69302.

18. Ishimaru, K., Miyagawa, S., Fukushima, S., Saito, A., Sakai, Y., Ueno, T., and Sawa, Y. (2013). Synthetic prostacyclin agonist, ONO1301, enhances endogenous myocardial repair in a hamster model of dilated cardiomyopathy: a promising regenerative therapy for the failing heart. J. Thorac. Cardiovasc. Surg. *146*, 1516–1525.

19. Shirasaka, T., Miyagawa, S., Fukushima, S., Saito, A., Shiozaki, M., Kawaguchi, N., Matsuura, N., Nakatani, S., Sakai, Y., Daimon, T., et al. (2013). A slow-releasing form of prostacyclin agonist (ONO1301SR) enhances endogenous secretion of multiple cardiotherapeutic cytokines and improves cardiac function in a rapid-pacing-induced model of canine heart failure. J. Thorac. Cardiovasc. Surg. *146*, 413–421.

20. Kubota, Y., Miyagawa, S., Fukushima, S., Saito, A., Watabe, H., Daimon, T., Sakai, Y., Akita, T., and Sawa, Y. (2014). Impact of cardiac support device combined with slow-release prostacyclin agonist in a canine ischemic cardiomyopathy model. J. Thorac. Cardiovasc. Surg. *147*, 1081–1087.

21. Watanabe, Y., Miyagawa, S., Fukushima, S., Daimon, T., Shirakawa, Y., Kuratani, T., and Sawa, Y. (2014). Development of a prostacyclin-agonist-eluting aortic stent graft enhancing biological attachment to the aortic wall. J. Thorac. Cardiovasc. Surg. *148*, 2325–2334.e1.

22. Yajima, S., Miyagawa, S., Fukushima, S., Sakai, Y., Isohashi, K., Watabe, T., Ikeda, H., Horitsugi, G., Harada, A., Sakaniwa, R., et al. (2018). A prostacyclin agonist and an omental flap increased myocardial blood flow in a porcine chronic ischemia model. J. Thorac. Cardiovasc. Surg. *156*, 229–241.e14.

23. Fukushima, S., Miyagawa, S., Sakai, Y., and Sawa, Y. (2015). A sustained-release drug-delivery system of synthetic prostacyclin agonist, ONO-1301SR: a new reagent to enhance cardiac tissue salvage and/or regeneration in the damaged heart. Heart Fail. Rev. *20*, 401–413.

24. Uchida, T., Hazekawa, M., Yoshida, M., Matsumoto, K., and Sakai, Y. (2013). Novel long-acting prostacyclin agonist (ONO-1301) with an angiogenic effect: promoting synthesis of hepatocyte growth factor and increasing cyclic AMP concentration via IP-receptor signaling. J. Pharmacol. Sci. *123*, 392–401.

25. Rigato, M., Monami, M., and Fadini, G.P. (2017). Autologous cell therapy for peripheral arterial disease: systematic review and meta-analysis of randomized, nonrandomized, and noncontrolled studies. Circ. Res. *120*, 1326–1340.

26. Pola, R., Gaetani, E., Flex, A., Aprahamian, T.R., Bosch-Marcé, M., Losordo, D.W., Smith, R.C., and Pola, P. (2004). Comparative analysis of the in vivo angiogenic properties of stable prostacyclin analogs: a possible role for peroxisome proliferator-activated receptors. J. Mol. Cell. Cardiol. *36*, 363–370.

27. Hoeben, A., Landuyt, B., Highley, M.S., Wildiers, H., Van Oosterom, A.T., and De Bruijn, E.A. (2004). Vascular endothelial growth factor and angiogenesis. Pharmacol. Rev. *56*, 549–580.

28. Morishita, R., Nakamura, S., Nakamura, Y., Aoki, M., Moriguchi, A., Kida, I., Yo, Y., Matsumoto, K., Nakamura, T., Higaki, J., and Ogihara, T. (1997). Potential role of an endothelium-specific growth factor, hepatocyte growth factor, on endothelial damage in diabetes. Diabetes *46*, 138–142.

29. Zernecke, A., Schober, A., Bot, I., von Hundelshausen, P., Liehn, E.A., Möpps, B., Mericskay, M., Gierschik, P., Biessen, E.A., and Weber, C. (2005). SDF-1α/CXCR4 axis is instrumental in neointimal hyperplasia and recruitment of smooth muscle progenitor cells. Circ. Res. *96*, 784–791.

30. Yamaguchi, J., Kusano, K.F., Masuo, O., Kawamoto, A., Silver, M., Murasawa, S., Bosch-Marce, M., Masuda, H., Losordo, D.W., Isner, J.M., and Asahara, T. (2003). Stromal cell-derived factor-1 effects on ex vivo expanded endothelial progenitor cell recruitment for ischemic neovascularization. Circulation *107*, 1322–1328.

31. Burkholder, T.J. (2007). Mechanotransduction in skeletal muscle. Front. Biosci. *12*, 174–191.

32. Nishida, H., Yamaguchi, T., Sato, K., Ohtsu, H., and Akiba, Y. (1997). Influence of prostaglandin E2 on chick myoblasts differentiation during the myogenesis. Anim. Sci. Technol. (Jpn) *68*, 381–384.

33. Tatsumi, R., Hattori, A., Ikeuchi, Y., Anderson, J.E., and Allen, R.E. (2002). Release of hepatocyte growth factor from mechanically stretched skeletal muscle satellite cells and role of pH and nitric oxide. Mol. Biol. Cell *13*, 2909–2918.

34. Zammit, P.S., Relaix, F., Nagata, Y., Ruiz, A.P., Collins, C.A., Partridge, T.A., and Beauchamp, J.R. (2006). Pax7 and myogenic progression in skeletal muscle satellite cells. J. Cell Sci. *119*, 1824–1832.

OMTM, Volume *18*

Supplemental Information

Administration of Slow-Release Synthetic Prostacyclin Agonist Promoted Angiogenesis and Skeletal Muscle Regeneration for Limb Ischemia

Takaya Nakagawa, Shigeru Miyagawa, Takashi Shibuya, Yoshiki Sakai, Akima Harada, Kenichi Watanabe, and Yoshiki Sawa

Supplement data 1

Sline	preoperation	postoperation	1 week	2 week	3 week	4 week
①	95.9	9.9	21.6	31	26.3	36.1
②	116.9	8	12.0	21.8	29	31.4
③	85.9	8.2	22.9	28.4	25.2	30.5
④	87.1	3.3	7.59	23.9	25.2	26
⑤	91.6	5.5	7.0	15.1	33.8	32.8
⑥	86.5	5.76	19.6	21.3	26.4	29
⑦	111.4	3.11	13.6	37.6	38.4	38.6
⑧	107.1	6.58	13.1	22.5	35.2	33.6
⑨	127.5	7.88	12.4	21.15	25.5	26.3
⑩	97.6	3.3	8.69	23	24.9	27.1
average	100.75	6.153	13.848	24.575	28.99	31.14
standard deviation	13.62793088	2.25668806	5.42137584	5.939160294	4.70987261	4.003548426

Supplement data 2

YS-1402	preoperation	postoperation	1 week	2 week	3 week	4 week
①	105.3	4.19	10.9	29.5	36.7	43.8
②	101.2	4.4	17.7	23.9	35.8	45.9
③	81.6	4.2	11.0	13.2	68.7	75.1
④	79	9.57	21.5	42.5	43.2	50.4
⑤	94.8	3.91	3.14	16.7	35.7	43.1
⑥	128	5.18	27	32.5	49.1	63
⑦	135	4.14	14.2	18.96	33.3	51.7
⑧	100	2.82	6.66	25.3	32.1	46.7
⑨	91.3	4.05	17.9	56.6	71.7	81.9
average	101.8	4.717777778	14.44444444	28.79555556	45.14444444	55.73333333
standard deviation	17.92471912	1.80859539	7.014567558	12.89946348	14.28970967	13.48900787

Supplement data 3

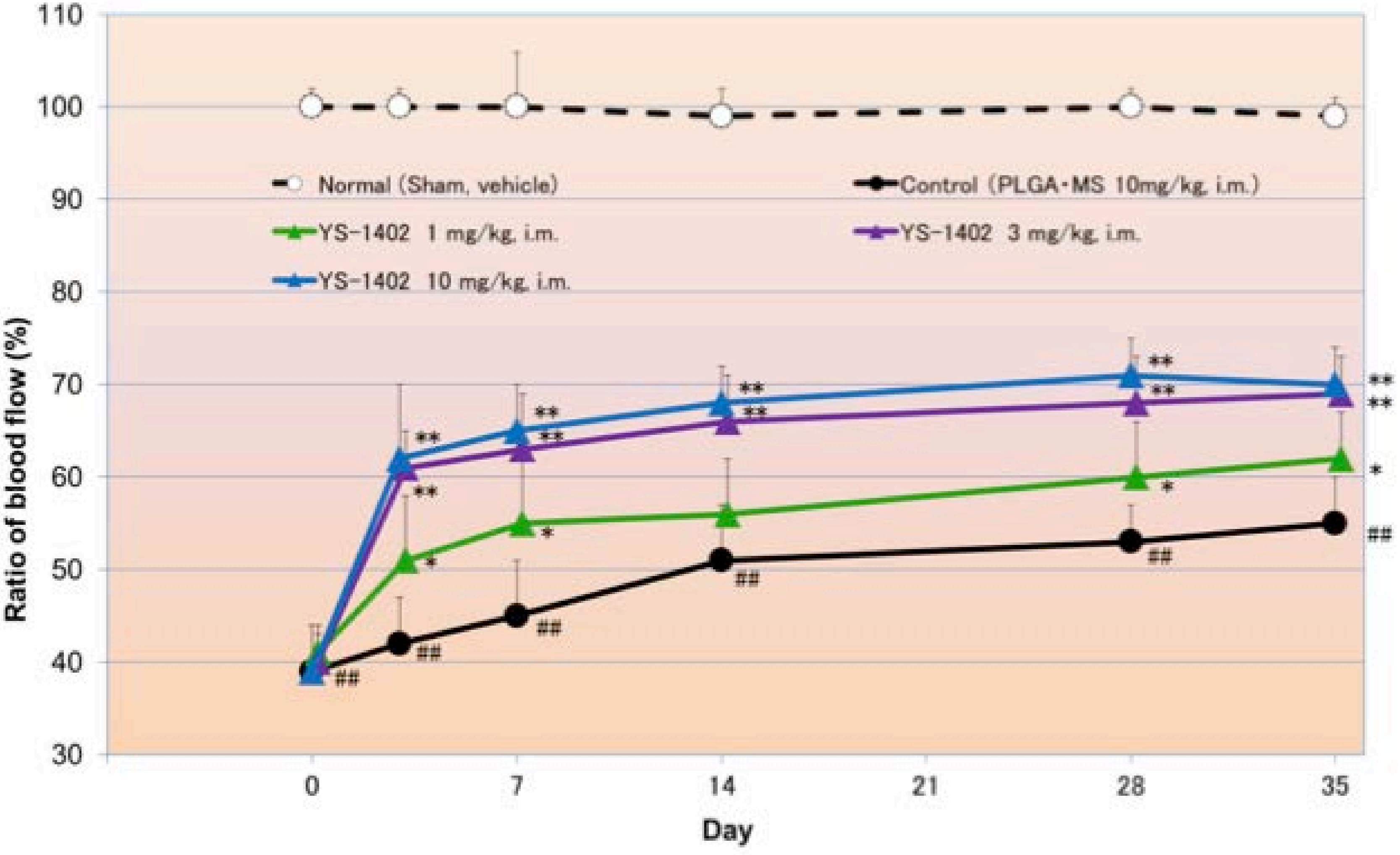

Supplement data 4

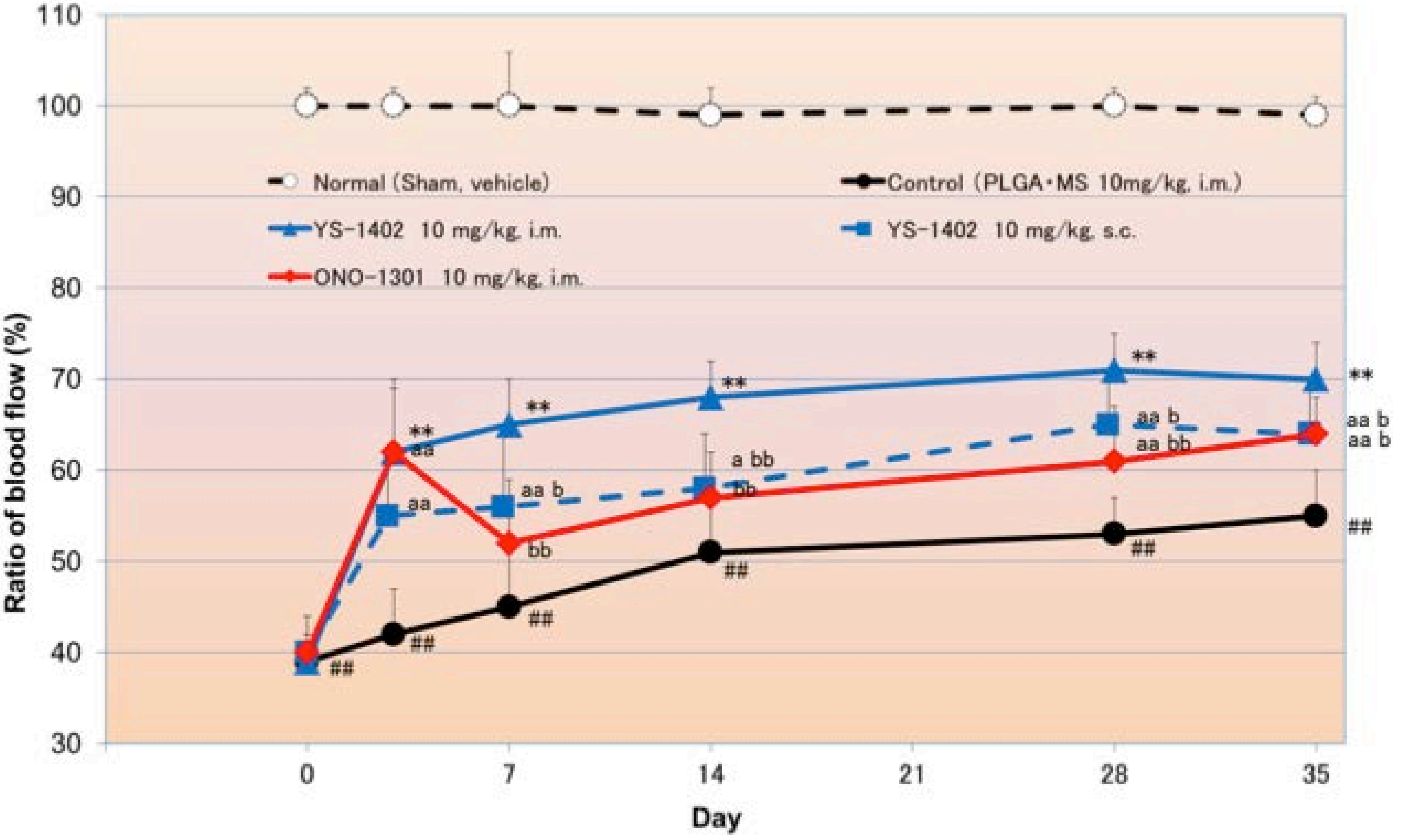

Supplement: Document S2. Article plus Supplemental Information [file mmc2.pdf]
